# Comparison of Statistical and Clinical Predictions of Functional Outcome after Ischemic Stroke

**DOI:** 10.1371/journal.pone.0110189

**Published:** 2014-10-09

**Authors:** Douglas D. Thompson, Gordon D. Murray, Cathie L. M. Sudlow, Martin Dennis, William N. Whiteley

**Affiliations:** 1 Centre for Population Health Sciences, University of Edinburgh, Edinburgh, United Kingdom; 2 Centre for Clinical Brain Sciences, University of Edinburgh, Edinburgh, United Kingdom; Harvard Medical School, United States of America

## Abstract

**Background:**

To determine whether the predictions of functional outcome after ischemic stroke made at the bedside using a doctor’s clinical experience were more or less accurate than the predictions made by clinical prediction models (CPMs).

**Methods and Findings:**

A prospective cohort study of nine hundred and thirty one ischemic stroke patients recruited consecutively at the outpatient, inpatient and emergency departments of the Western General Hospital, Edinburgh between 2002 and 2005. Doctors made informal predictions of six month functional outcome on the Oxford Handicap Scale (OHS). Patients were followed up at six months with a validated postal questionnaire. For each patient we calculated the absolute predicted risk of death or dependence (OHS≥3) using five previously described CPMs. The specificity of a doctor’s informal predictions of OHS≥3 at six months was good 0.96 (95% CI: 0.94 to 0.97) and similar to CPMs (range 0.94 to 0.96); however the sensitivity of both informal clinical predictions 0.44 (95% CI: 0.39 to 0.49) and clinical prediction models (range 0.38 to 0.45) was poor. The prediction of the level of disability after stroke was similar for informal clinical predictions (ordinal c-statistic 0.74 with 95% CI 0.72 to 0.76) and CPMs (range 0.69 to 0.75). No patient or clinician characteristic affected the accuracy of informal predictions, though predictions were more accurate in outpatients.

**Conclusions:**

CPMs are at least as good as informal clinical predictions in discriminating between good and bad functional outcome after ischemic stroke. The place of these models in clinical practice has yet to be determined.

## Introduction

Stroke patients, their families and their doctors would like an accurate prediction of disability or death (poor functional outcome) in the short and medium-term. Most predictions of poor functional outcome in stroke patients are made informally, based upon the clinical experience of doctors looking after them. However, statistical models, which calculate the probability of poor functional outcome based upon weights given to different clinical variables, may make more accurate predictions. Previous studies comparing the predictions of models and clinical opinion after stroke have either only examined one prediction model [1] or have examined predictions based on case scenarios, rather than face to face [2].

In making predictions about individual events, statistical models with a few simple variables perform similarly to experts [3]. However, in clinical practice it is a common belief that a doctor's intuition, or the use of more complex models, lead to more accurate and acceptable predictions.

We sought to determine whether the predictions of poor functional outcome made at the bedside using a doctor’s clinical experience were better or worse than the predictions made by statistical models in patients with first recent ischemic stroke.

## Methods

### Ethics statement

The study was approved by the Lothian Research Ethics Committee. All patients or their relatives provided written informed consent for the collection of samples and subsequent analysis.

The Edinburgh Stroke Study (ESS) recruited consecutive patients after a first recent stroke from the outpatient, inpatient and emergency departments of the Western General Hospital, Edinburgh between April 2002 and May 2005 (www.dcn.ed.ac.uk/ess/protocol) [4]. We measured informal clinical predictions (‘gestalt’) by asking doctors with varying levels of experience in stroke medicine to predict the six month Oxford Handicap Scale (OHS) (a widely used variant of the modified Rankin Scale) in patients at presentation using their clinical experience. We classified doctors by seniority (fully trained in neurology or stroke medicine versus in training) and parent speciality (geriatrics/internal medicine versus neurology). Doctors measured baseline clinical variables which were used in the clinical prediction rules with a standardised pro-forma. Blind to baseline characteristics and independent from clinicians who initially assessed patients, we measured functional outcome with the OHS at six months using a validated postal questionnaire, and sent a repeat questionnaire to non-responders. All patients were ‘flagged’ for death with the General Register Office for Scotland, which provided information on the date, place and cause of death. We defined ‘poor functional outcome’ at six months as OHS≥3 (i.e., dead or dependent on others for activities of daily living). We restricted this analysis to patients with definite or probable ischemic stroke, which was defined as a focal deficit of cerebral origin lasting for ≥24 hours, where brain imaging showed either positive evidence of cerebral infarction, or was normal or equivocal and the clinical syndrome was most in keeping with stroke.

### Predicting poor functional outcome using pre-existing statistical models

We identified statistical prediction models from a previously published systematic review of models for risk of poor functional outcome after stroke and identified five multivariable binary logistic regression models (Table 1) [5]. We calculated the linear predictor (a linear combination of risk factors individually multiplied by an associated coefficient) for each of these models with the reported regression coefficients, or else the natural logarithm of the odds ratios.

**Table 1:** Formal statistical prediction models for functional outcome.

Variables	Lee et al[21]	Appelros et al[22]	Weimar et al[23], [24]	Counsell et al(SSV) [8]	Reid et al[25]
Intercept			−5.782	+12.340	+2.401
Age		+0.077	+0.049	−0.051	−0.049
Pre-strokeindependence				−2.744	+3.497
Living alone				+0.661	
Arm power				−2.106	+1.402
Able to walk				−1.311	
Normal GCSverbal				−2.160	
NIHSS(strokeSeverityscore)	+0.362	+0.285	+0.272		−0.549
Heart failure		+1.099			
History ofdiabetes	−2.296				
Totalcholesterol	−0.029				
Outcome	mRS>2 at sixmonths	mRS≥3 atone year	BI<95 ordead	OHS≤2 at sixmonths	OHS≤2 at sixmonths
Sourcepopulation	Taiwanesehospitalcohort	Communitybased cohortof first everstrokes inSweden	Stroke databank of theGermanStrokeFoundation	OCSPcommunitybasedincidencestudy	Consecutivepatientsenrolled inthe StrokeOutcomeStudy
Additionalcomments		Coefficientsestimatedfrom thenatural log ofodds ratiosreported totwo decimalplaces		The ‘SSV’model. Scores1 for presence and2 for absenceof risk factor	Strokeseverity wasmeasuredusing a scoreadapted fromthe EC/ICbypass study

NOTE: Individual beta coefficients from each model (NB: +/− values indicate an increase/decrease in the log-odds of outcome). Some models predicted poor outcomes 21,22,23,24] others predicted good outcomes 8,25], as the latter is the inverse of the former, all can be used to predict good or poor outcomes. ABBREVIATIONS: modified Rankin Scale (mRS); the Oxford Handicap Scale (OHS); National Institutes of Health Stroke Scale (NIHSS); Glasgow Comma Scale (GCS); Barthel Index (BI); Six Simple Variables model (SSV); and the Oxford Community Stroke Project classification (OCSP).

### Accuracy of formal versus informal prediction: a dichotomous outcome

**Figure 1:**
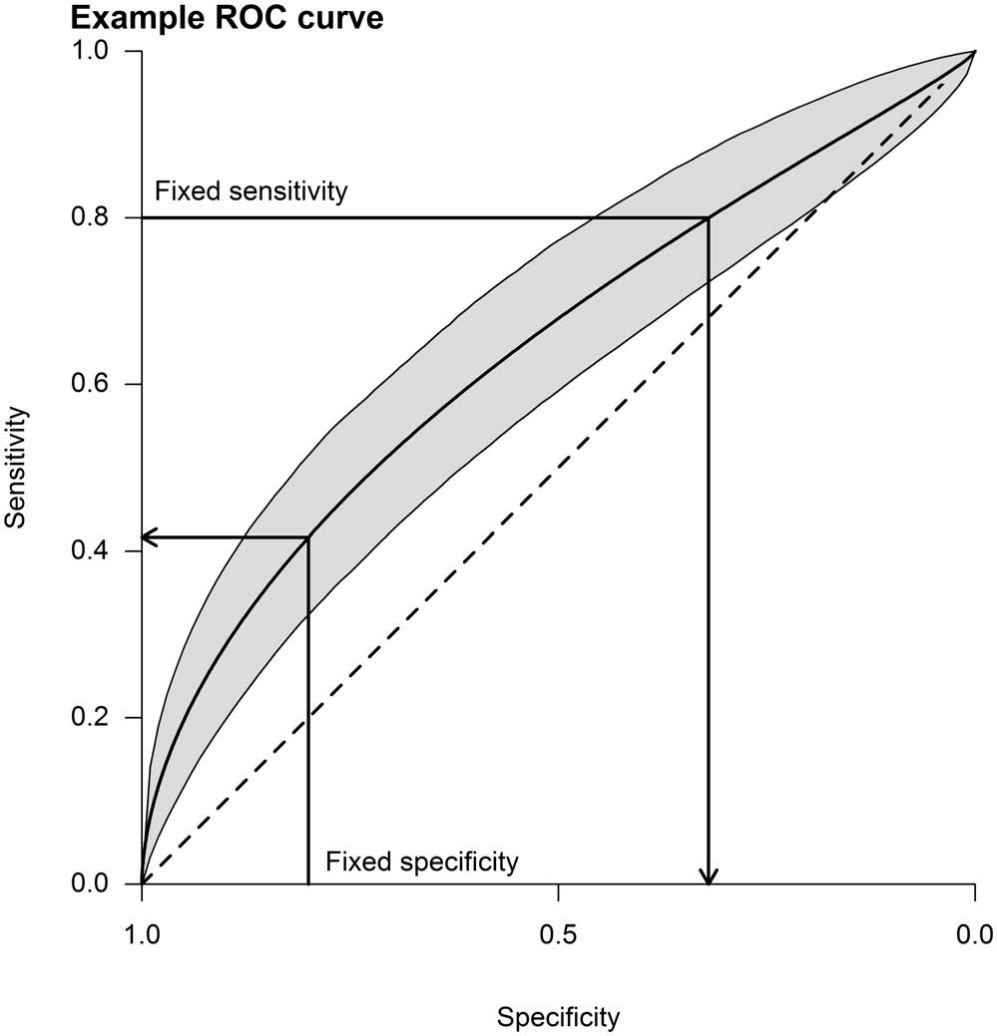
Example Receiver Operating Characteristic (ROC) curve. Note: the threshold at fixed specificity/sensitivity achieved by doctors are used to calculate the corresponding sensitivity/specificity of the prediction model. The shaded area shows the 95% confidence interval about the ROC curve.

We calculated the thresholds of predicted probability of poor functional outcome (OHS≥3) for each model that had: (i) the same specificity; and (ii) the same sensitivity as the doctor’s predictions. We then calculated the model sensitivity at the threshold of doctors’ specificity and model specificity at the threshold of doctors’ sensitivity (Figure 1). We estimated 95% confidence intervals (CI) for sensitivities and specificities using 1000 bootstrap replicates for clinical prediction models and used 95% Zhou-Li (ZL) confidence intervals for doctors informal predictions [6]. The area under the receiver operating characteristic curve (AUROCC) is a standard measure for assessing model discrimination for a binary outcome. Given two randomly selected patients, one with poor functional outcome in follow-up and one without, the AUROCC is the probability that the model assigns a greater risk to the patient with the event. It ranges from no better than chance (0.5) to perfect (1.0) discrimination [7]. We assessed model calibration by plotting observed outcomes against predicted risk in equal groups; a perfectly calibrated model would fit a line with slope 1 and intercept 0. We undertook a sensitivity analyses stratifying by where patients were seen since one of the five models was reported to discriminate poor functional outcome less well in outpatients [8].

### Accuracy of formal versus informal prediction: an ordinal outcome

We analysed the data with the complete range of the OHS to explore whether clinical prediction models and doctors informal predictions could predict the level of disability or death after stroke. We used the weighted kappa (κ) statistic (with squared error weights) to assess agreement between a doctor’s informal prediction of the six month OHS with the observed six month OHS. The weighted kappa adjusts for chance agreement and ranges from no agreement (0) to perfect agreement (1). We used ordinal logistic regression with observed OHS as the outcome and compared a model where clinicians’ informal prediction was the only predictor to models taking the individual linear predictors from formal prediction as the only predictor. The ESS comprises a mixture of inpatients and outpatients. Fewer patients with higher OHS scores were expected in outpatients [1]. To ensure the identifiability of parameter estimates we analysed the OHS as a five level measure collapsing 4, 5, and 6 to leave levels 0, 1, 2, 3 and ≥4. We measured discrimination with the ordinal *c*-index (ORC), a set-based measure which summarises how close the predicted ordering is to the observed. This is interpreted as the probability that given two patients with differing levels of an observed outcome a prediction model assigns a greater risk to the patient with the worse outcome [9]. We calculated 95% CIs for each ORC measure using 1000 bootstrap replicates.

Doctors’ informal predictions were categorised as: correct (observed OHS matches predicted); optimistic (observed OHS is greater than predicted); or pessimistic (observed OHS is less than predicted). We investigated the effect of doctors’ experience adjusted for patient characteristics with binary logistic regression to ascertain which patients were most likely to be optimistically classified. We carried out sensitivity analysis using multinomial logistic regression [10] to account for all three potential outcomes.

An analysis including only cases with complete baseline data reduces statistical power and may introduce bias. We imputed missing baseline data generating 20 datasets. We used six month OHS in our imputation model but removed those with missing follow-up from all analysis since retaining imputed outcomes would only add random noise to our results [11]. We performed sensitivity analyses for: patients who were admitted to hospital and those who were seen as outpatients; for the OHS dichotomised at ≥2; and for periods of delay from stroke onset to assessment, namely <2 days, 2 to 7 days and >7 days.

We carried out all analyses using R version 3.0.1 with add-on packages rms, pROC, nnet and simpleboot.

## Results

The patients available for analysis are summarised in Table 2 and Figure S1. Of 1257 patients (671 outpatients and 586 inpatients), 1051 (84%) had record of doctor’s predictions of which 931 had complete follow-up by six months. On average those with missing six month outcomes or missing doctors’ predictions were younger (median 73 years versus 74 years, P-value = 0.0130) and had less severe strokes (median NIHSS of 1 versus 2, P-value = 0.0193) (see Table S1). Outpatients were more likely to have a missing informal prediction made by a clinician (20% versus 13%, P-value = 0.0014). At six months 603 (65%) of the 931 patients had a good functional outcome (OHS of 0, 1 or 2) and 98 (11%) had died. The median time from stroke onset to assessment for inpatients was 2 days (with interquartile range (IQR) 1 to 4) and for outpatients was 18 days (IQR 12 to 28). These data are limited by consent bias. Of those that were eligible, 88% consented for their data to be part of a repository for further research, the main barrier to which was obtaining informed consent [12]. Despite this, characteristics were similar between those consenting and non-consenting patients.

**Table 2:** Characteristics of 931 ischemic stroke patients observed in the ESS.

Variable	Data	Number (%) missing
**Doctor’s experience**		
Fully trained versus in training, n (%)	499 (54)	107 (11)
Geriatrics/internal medicine specialist versus neurologist, n (%)	550 (59)	107 (11)
**Baseline characteristics**		
Age, years, median (IQR)	74 (66 to 81)	-
Male, n (%)	474 (51)	-
History of hypertension, n (%)	520 (56)	1 (<1)
History of diabetes mellitus, n (%)	119 (13)	-
Pre-stroke independence, n (%)	867 (93)	2 (<1)
Lived alone prior to stroke, n (%)	361 (39)	-
Arm power, n (%)	799 (86)	1 (<1)
Able to walk, n (%)	672 (72)	2 (<1)
Normal GCS verbal, n (%)	810 (87)	5 (<1)
NIHSS (median, IQR)	2 (0 to 5)	35 (4)
Heart failure, n (%)	55 (6)	2 (<1)
Total cholesterol, mmol/l, median (IQR)	5 (4 to 6)	73 (8)
Systolic BP, mmHg, median (IQR)	146 (130 to 160)	2 (<1)
Seen at outpatients, n (%)	489 (53)	-
**Six month OHS score, n (%)**		
0 (Fully recovered)	168 (18)	-
1	252 (27)	-
2	183 (20)	-
3	126 (14)	-
4	49 (5)	-
5	55 (6)	-
6 (Dead)	98 (11)	-

ABBREVIATIONS: Oxford Handicap Scale (OHS); National Institutes of Health Stroke Scale (NIHSS); Glasgow Comma Scale (GCS); Inter Quartile Range (IQR); and Blood Pressure (BP).

### Doctor’s informal prediction versus formal statistical prediction

Eighteen doctors made clinical predictions: ten neurologists (56%) and eight stroke physicians (44%). Ten were in training (56%) and eight were fully trained (44%). Doctors correctly predicted level of disability or death in 310/931 patients (33%). Doctor’s informal predictions of poor functional outcome (i.e., OHS≥3) six months after stroke had a sensitivity of 0.44 (95% 0.39 to 0.49) and a specificity of 0.96 (95% 0.94 to 0.97). The performance of clinical prediction models was similar: at the specificity of a doctor (0.96), the sensitivity of risk prediction rules to predict poor functional outcome ranged from 0.38 to 0.45; at the sensitivity of a doctor (0.44) specificity of risk prediction rules ranged from 0.94 to 0.96 (Table 3). There were no important differences in these results when defining poor functional outcome as OHS≥2 rather than OHS≥3 (Table S2 and Table S3).

**Table 3:** Performance of formal and informal prediction on a dichotomous split (OHS≥3) and across an ordinal OHS (defined on five levels: 0, 1, 2, 3 and ≥4).

	Dichotomous outcome: OHS≥3		Ordinal outcome
Method of prediction	Sensitivity	Specificity	ORC
Doctor	0.44 (0.39 to 0.49)	0.96 (0.94 to 0.97)	0.74 (0.72 to 0.76)
Statistical model			
Reid [25]	0.45 (0.34 to 0.52)	0.96 (0.93 to 0.98)	0.75 (0.73 to 0.77)
Weimar [23]	0.43 (0.35 to 0.51)	0.96 (0.92 to 0.98)	0.73 (0.71 to 0.76)
SSV [8]	0.43 (0.36 to 0.51)	0.95 (0.93 to 0.98)	0.72 (0.70 to 0.74)
Appelros [22]	0.42 (0.35 to 0.50)	0.95 (0.93 to 0.97)	0.73 (0.71 to 0.75)
Lee [21]	0.38 (0.32 to 0.45)	0.94 (0.91 to 0.96)	0.69 (0.66 to 0.71)

NOTE: The ORC is a measure of discrimination for ordinal models ranging from 0.5 (no discrimination) to 1 (perfect discrimination). ABBREVIATIONS: Ordinal *c*-index (ORC); Oxford Handicap Scale (OHS) and the Six Simple Variables model (SSV). Note that all confidence intervals are 95% CIs. The ORC (an ordinal equivalent to the AUROCC) and the sensitivities/specificities CIs are calculated over 1000 bootstrap replicates within a single imputation of the ESS and the doctors’ sensitivity and specificity CIs are Zhou-Li intervals.

Clinical prediction models had similar overall discrimination for poor functional outcome (OHS≥3) to one another, and all discriminated moderately well (AURCOCCs ranging from 0.76 to 0.84 see Table S3) despite the differing outcomes they were originally developed to predict. Model calibration, where it could be assessed, was poor. Each model systematically underestimated the risk of poor outcome (calibration intercept >0) except for the six simple variables model, which over predicted patient risk (calibration intercept <0). In a sensitivity analysis, there was no important improvement in calibration in hospital inpatients or when restricting to early and late delay from stroke onset to assessment, though there was evidence to suggest poorer discrimination (AUROCCs<0.75) in those with later assessment and those seen as outpatients (Table S3 and Table S4).

Doctor’s predictions of OHS at six months agreed moderately with the observed six month OHS for inpatients (weighted κ of 0.53 with 95% CI 0.42 to 0.63) but were poor for outpatients (0.30 with 95% CI 0.21 to 0.39). Doctors tended toward optimistic prediction with 61% (95% CI 55% to 68%) of inpatients and 45% (95% CI 38% to 51%) of outpatients given a lower predicted OHS than observed. Ordinal discrimination by doctors was moderate (ORC of 0.74 with 95% CI 0.72 to 0.76) and comparable to clinical prediction models, which ranged from 0.69 to 0.75 (Table 3). The results were similar in inpatients but there was worse discrimination in outpatients. Clinical prediction models therefore did no better than doctors’ informal prediction of level of disability after stroke.

**Figure 2:**
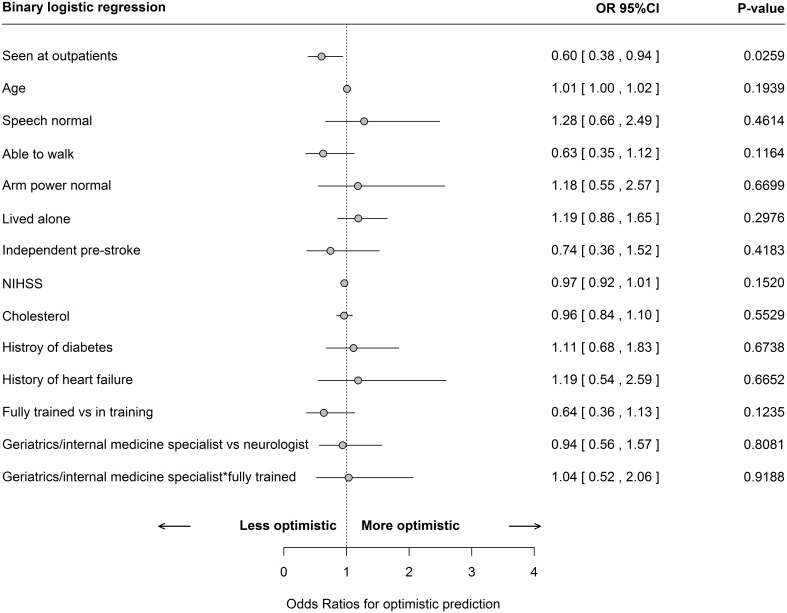
Multivariable binary logistic regression model comparing optimistic prediction to those correctly classified by doctors. Note: An interaction is denoted by an asterisk. Data are on a single imputed set and are restricted to those patients for whom doctors’ characteristics could be obtained (N = 700 patients, of which: 282 where correctly classified and 418 optimistically classified).

We were unable to demonstrate that doctor (level of training or speciality) or patient characteristics (neurological impairment, age or risk factors) led to over-optimistic predictions of poor functional outcome (Figure 2). However, patients seen in an outpatient setting were more likely to have had a correct prediction of their eventual disability than those seen as inpatients (OR of 0.60 with 95% CI 0.38 to 0.94), probably as most were assessed relatively late. No quantitative differences were found when modelling pessimistic classification versus optimistic classification with multinomial logistic regression (Figure S2).

## Discussion

In this study, the accuracy of prediction of poor functional outcome after stroke was similar whether made by a doctor’s informal prediction or by formal statistical prediction. For the prediction of poor functional outcome after stroke both methods had a good specificity, but a poor sensitivity. There was no evidence that characteristics of doctors or patients made a great deal of difference to the accuracy of informal predictions, but predictions tended to be more likely to be correct in outpatients. This is likely due to the delay in onset to assessment (18 days with IQR 12 to 28) by which point most patients suffering minor strokes would have recovered, making their observed disability at outpatients a good surrogate for their likely disability by six month.

Our study has a number of strengths. We studied predictions made by doctors in the course of their clinical practice, rather than in simulation studies or made retrospectively on routinely collected data, and compared them to model predictions made with variables also collected in the course of clinical practice. Our conclusions are likely to be applicable to clinical settings similar to ours. We examined predictions in a prospective cohort, and measured outcome at a clinically relevant time-point after stroke. Unlike previous analyses, we examined the whole range of the OHS, and found that both clinicians and statistical models were able to make reasonable predictions of the level of six month disability. Doctors may find it easier to predict grouped disability rather than individual levels of the OHS. We explored the two most commonly adopted dichotomies of the OHS; though this made little impact to the ordering of the formal methods of prediction which remained similar to that of a doctor.

Our analyses were limited by missing data, which we sought to mitigate by imputation of missing baseline data. However, it is possible that performance of clinical prediction models and informal clinician prediction in those patients who could not be analysed differs to an important degree from those patients who we included. We were unable to examine more recently developed clinical prediction models, such as the ASTRAL score [13], the iScore [14] and BOAS [15]) as not all of the baseline predictors of these models were available in the Edinburgh Stroke Study. It is therefore possible that one of these models might perform better in this dataset than the other models or clinical prediction, but we believe that this is not likely, as the performance of the models in their development cohorts and subsequent validation studies was similar to the performance of the other clinical prediction models evaluated in our study [13], [15], [16], [17]. These models required different measures of deficits caused by the stroke (e.g., the use of the Canadian Neurological Scale) or else a record of various co-morbidities (e.g., cancer or renal dialysis). For the most part these characteristics were well represented within those models we could test and it is unlikely that the inclusion of these variables would result in any considerable improvement. We therefore expect that these models would rank in a similar way with respect to doctors predictions. Some of the prediction models we tested were developed to predict distinct outcomes or used predictors with definitions that differed to those used in our analysis. Specifically, the model developed by Reid et al included a stroke severity score which - though useful - is rarely used in practice and was therefore not available in our data [18]. We used the NIHSS in its place and found that regardless of the qualitative differences in these variables the Reid model performed well in our data. This is likely due to the strong correlation between the stroke severity score and the NIHSS [19]. Our findings are therefore supportive of a strong degree of generalisability in the discriminatory ability of these models; though updating would likely be required to improve upon calibration [20]. We did not adjust or update these models to account for any differences between the development and evaluation settings (i.e., differences in baseline characteristics or outcome definition etc.); despite this model performance was good in our data. Our conclusion may be limited to institutions like ours: it may be that outside of different hospitals, the relative performance of model-based predictions versus informal clinical prediction is different. Additionally, the majority of patients in the ESS had mild strokes. It is therefore possible that the scores would have a different performance in a population of more severe stroke patients, though the relative performance of the models and doctors discrimination is unlikely to change.

Previous studies have conflicting findings; a simulation study demonstrated that doctor’s predictions were worse than model-based predictions, albeit based on scenario-based, rather than clinical predictions [2]. A similar study to ours, demonstrated that doctor’s predictions of poor functional outcome were similar to the six simple variables model [1].

Clinical prediction models for predicting poor prognosis after stroke have yet to find a place in clinical practice. It seems reasonably clear that clinical prediction models make predictions of poor functional outcome that are at least as good as informal predictions made by doctors. Whilst the inclusion of more complex variables such as NIHSS and stroke subtype have intuitive appeal, they add to the difficulty of using models, which may limit their use by non-specialists, non-doctors, and doctors early in their training. It is unclear whether model–based predictions are more acceptable to patients or lead to better decisions about clinical care, rehabilitation, or targeting of resources. We believe that there would be merit in conducting an impact study of any one of these models to assess what benefit there is in adopting model-based risk predictions in clinical practice.

In conclusion, clinical prediction models are at least as accurate as informal clinical predictions in determining the risk of poor functional outcome after ischemic stroke. The place of these models in clinical practice has yet to be determined.

## Supporting Information

Figure S1 **Flowchart of data available for analysis in the ESS.**
(TIFF)

Figure S2 **Multinomial logistic regression model of doctors classification of patients.** Solid points denote the classifications ‘optimistic vs. correct’ and open points denote ‘pessimistic vs. correct’.
(TIFF)

Table S1 **Prevalence of risk factors at baseline in those included in analysis versus those with either missing informal prediction or missing observed outcome at six month follow-up.**
(DOC)

Table S2 **Prediction of poor outcome (OHS≥2) following stroke for inpatients (N = 442), outpatients (N = 489) and all patients (N = 931).**
(DOC)

Table S3 **Prediction of poor outcome (OHS≥3) following stroke for inpatients (N = 442), outpatients (N = 489) and all patients (N = 931).**
(DOC)

Table S4 **Prediction of poor outcome (OHS≥3) following stroke for patients assessed within: 2 days (N = 197); 2 to 7 days (N = 234) and over 7 days (N = 500) from stroke onset.**
(DOC)
