# Supplementary figures and images for: Comparison of Statistical and Clinical Predictions of Functional Outcome after Ischemic Stroke
Source: PLoS One. 2014 Oct 9;9(10):e110189. doi: 10.1371/journal.pone.0110189 (PMC4192583; doi:10.1371/journal.pone.0110189)

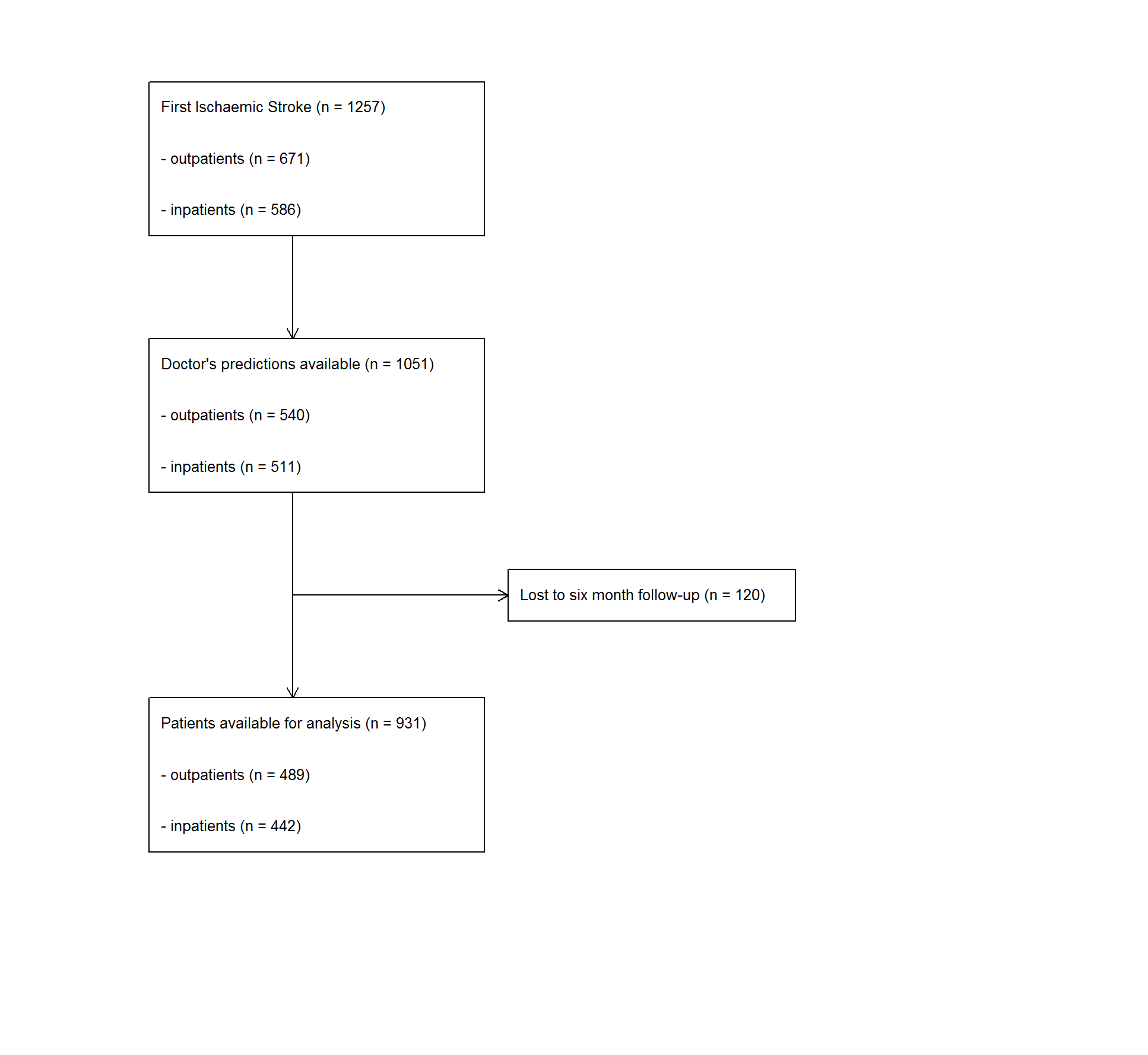

Supplement: Figure S1 — Flowchart of data available for analysis in the ESS. (TIFF) [file pone.0110189.s001.tiff]
