# Supplementary material for: Comparison of Statistical and Clinical Predictions of Functional Outcome after Ischemic Stroke
Source: PLoS One. 2014 Oct 9;9(10):e110189. doi: 10.1371/journal.pone.0110189 (PMC4192583; doi:10.1371/journal.pone.0110189)
Supplement: Table S1 — Prevalence of risk factors at baseline in those included in analysis versus those with either missing informal prediction or missing observed outcome at six month follow-up. (DOC) [file pone.0110189.s003.doc]

Table S1 - Prevalence of risk factors at baseline in those included in analysis versus those with either missing informal prediction or missing observed outcome at six month follow-up.

|  | **Patients available for analysis (n = 931)** | |  | **Missing outcome or prediction (n = 326)** | |  |
| --- | --- | --- | --- | --- | --- | --- |
| **Measurements taken on entry** | **No. (%)** | **No. Missing** |  | **No. (%)** | **No. Missing** | **P-value** |
| **Variables used in formal prediction models** |  |  |  |  |  |  |
| Age (years) (median, IQR) | 74 (66 to 81) | - |  | 73 (61 to 81) | - | 0.0130 |
| Pre-stroke independence | 867 (93) | 2 |  | 298 (91) | 3 | 0.5167 |
| Lived alone prior to stroke | 361 (39) | - |  | 120 (37) | 2 | 0.5794 |
| Arm power | 799 (86) | 1 |  | 276 (85) | 2 | 0.7468 |
| Able to walk | 672 (72) | 2 |  | 240 (74) | 4 | 0.4445 |
| Normal GCS verbal | 810 (87) | 5 |  | 279 (86) | 3 | 0.6121 |
| NIHSS (median, IQR) | 2 (0 to 5) | 35 |  | 1 (0 to 3) | 42 | 0.0193 |
| Heart failure | 55 (6) | 2 |  | 25 (8) | 1 | 0.2619 |
| History of diabetes | 119 (13) | - |  | 39 (12) | - | 0.7012 |
| Total cholesterol (mmol/l) (median, IQR) | 5 (4 to 6) | 73 |  | 5 (4 to 6) | 46 | 0.3635 |
| **Additional variables** |  |  |  |  |  |  |
| Systolic BP (mmHg) (median, IQR) | 146 (130 to 160) | 2 |  | 148 (130 to 164) | 5 | 0.9778 |
| Stroke syndrome |  |  |  |  |  | 0.2853 |
| TACS | 97 (11) | - |  | 32 (10) | - | - |
| LACS | 250 (28) | - |  | 73 (24) | - | - |
| PACS | 412 (47) | - |  | 149 (49) | - | - |
| POCS | 122 (14) | - |  | 53 (17) | - | - |
| Missing | - | 50 |  | - | 19 | - |
| Gender, Male | 474 (51) | - |  | 170 (52) | - | 0.7012 |
| History of hypertension | 520 (56) | 1 |  | 163 (50) | - | 0.0653 |
| Atrial fibrillation | 66 (20) | 2 |  | 205 (22) | - | 0.4918 |
| Able to talk | 844 (91) | 1 |  | 295 (90) | 2 | 0.8734 |
| Seen at outpatients | 489 (53) | - |  | 182 (56) | - | 0.3036 |

NOTE: Data presented as number and percentage (%) unless otherwise stated. All P-values presented come from a Wald test, except for stroke syndrome which was from a Likelihood Ratio Test. ABBREVIATIONS: Total Anterior Circulation Stroke (TACS), Lacunar stroke (LACS), Partial Anterior Circulation Stroke (PACS), and Posterior Circulation Stroke (POCS).
