# Supplementary material for: Comparison of Statistical and Clinical Predictions of Functional Outcome after Ischemic Stroke
Source: PLoS One. 2014 Oct 9;9(10):e110189. doi: 10.1371/journal.pone.0110189 (PMC4192583; doi:10.1371/journal.pone.0110189)
Supplement: Table S2 — Prediction of poor outcome (OHS≥2) following stroke for inpatients (N = 442), outpatients (N = 489) and all patients (N = 931). (DOC) [file pone.0110189.s004.doc]

**Table S2 - Prediction of poor outcome (OHS≥2) following stroke for inpatients (N=442), outpatients (N=489**) and all patients (N=931)

|  |  | **Calibration** | |  | **Discrimination** | |  | **Fixed informal sensitivity/specificity^1^** | |
| --- | --- | --- | --- | --- | --- | --- | --- | --- | --- |
| **Patients** | **Median R^2^ (%, IQR)** | **Intercept** | **Slope** |  | **ORC** | **AUROCC** |  | **Sensitivity** | **Specificity** |
| **Inpatients** |  |  |  |  |  |  |  |  |  |
| Reid | 22.0 (21.5 to 22.3) | 2.50 (2.36 to 2.64) | 0.28 (0.24 to 0.33) |  | 0.71 (0.68 to 0.75) | 0.77 (0.72 to 0.82) |  | 0.60 (0.50 to 0.73) | 0.73 (0.64 to 0.83) |
| Weimar | 21.5 (20.9 to 21.7) | 1.96 (1.83 to 2.08) | 0.61 (0.52 to 0.71) |  | 0.71 (0.68 to 0.75) | 0.77 (0.72 to 0.82) |  | 0.60 (0.49 to 0.74) | 0.74 (0.64 to 0.82) |
| SSV | 24.0 (23.8 to 24.3) | 1.34 (1.21 to 1.48) | 0.53 (0.46 to 0.59) |  | 0.73 (0.70 to 0.76) | 0.78 (0.73 to 0.82) |  | 0.62 (0.51 to 0.71) | 0.72 (0.60 to 0.82) |
| Appelros^2^ | 22.9 (22.5 to 23.1) | - | - |  | 0.72 (0.68 to 0.75) | 0.77 (0.72 to 0.82) |  | 0.61 (0.53 to 0.75) | 0.74 (0.65 to 0.83) |
| Lee^2^ | 13.0 (12.8 to 13.3) | - | - |  | 0.66 (0.62 to 0.71) | 0.70 (0.65 to 0.75) |  | 0.54 (0.45 to 0.65) | 0.60 (0.47 to 0.72) |
| Doctor^3^ | - | - | - |  | 0.72 (0.69 to 0.77) | - |  | 0.69 (0.64 to 0.74) | 0.79 (0.70 to 0.85) |
| **Outpatients** |  |  |  |  |  |  |  |  |  |
| Reid | 9.9 (9.6 to 10.2) | 2.69 (2.59 to 2.79) | 0.60 (0.50 to 0.70) |  | 0.65 (0.61 to 0.70) | 0.66 (0.61 to 0.71) |  | 0.28 (0.20 to 0.34) | 0.86 (0.80 to 0.90) |
| Weimar | 6.9 (6.6 to 7.0) | 1.52 (1.43 to 1.62) | 0.73 (0.57 to 0.90) |  | 0.62 (0.58 to 0.66) | 0.63 (0.58 to 0.68) |  | 0.21 (0.13 to 0.29) | 0.80 (0.75 to 0.86) |
| SSV | 7.8 (7.8 to 7.8) | 0.80 (0.70 to 0.90) | 0.52 (0.41 to 0.63) |  | 0.60 (0.55 to 0.65) | 0.62 (0.56 to 0.67) |  | 0.24 (0.17 to 0.30) | 0.79 (0.71 to 0.85) |
| Appelros^2^ | 4.9 (4.7 to 4.9) | - | - |  | 0.61 (0.57 to 0.66) | 0.62 (0.57 to 0.67) |  | 0.19 (0.11 to 0.30) | 0.81 (0.72 to 0.87) |
| Lee^2^ | 0.1 (0.1 to 0.2) | - | - |  | 0.57 (0.51 to 0.62) | 0.57 (0.51 to 0.62) |  | 0.18 (0.11 to 0.28) | 0.80 (0.68 to 0.87) |
| Doctor^3^ | - | - | - |  | 0.65 (0.61 to 0.69) | - |  | 0.38 (0.31 to 0.45) | 0.92 (0.88 to 0.94) |
| **All patients** |  |  |  |  |  |  |  |  |  |
| Reid | 29.5 (29.3 to 30.0) | 2.62 (2.54 to 2.70) | 0.44 (0.40 to 0.48) |  | 0.75 (0.73 to 0.77) | 0.78 (0.75 to 0.81) |  | 0.56 (0.49 to 0.63) | 0.87 (0.83 to 0.90) |
| Weimar | 27.1 (26.9 to 27.2) | 1.68 (1.61 to 1.76) | 0.82 (0.74 to 0.90) |  | 0.73 (0.71 to 0.76) | 0.77 (0.74 to 0.80) |  | 0.54 (0.47 to 0.60) | 0.85 (0.79 to 0.90) |
| SSV | 26.7 (26.5 to 27.1) | 0.99 (0.92 to 1.07) | 0.64 (0.58 to 0.69) |  | 0.72 (0.70 to 0.74) | 0.75 (0.72 to 0.78) |  | 0.52 (0.46 to 0.58) | 0.80 (0.74 to 0.89) |
| Appelros^2^ | 26.5 (26.4 to 26.6) | - | - |  | 0.73 (0.71 to 0.75) | 0.76 (0.73 to 0.79) |  | 0.52 (0.44 to 0.58) | 0.83 (0.78 to 0.88) |
| Lee^2^ | 17.3 (17.2 to 17.4) | - | - |  | 0.69 (0.66 to 0.71) | 0.71 (0.67 to 0.74) |  | 0.47 (0.41 to 0.53) | 0.78 (0.72 to 0.84) |
| Doctor^3^ | - | - | - |  | 0.74 (0.72 to 0.76) | - |  | 0.58 (0.53 to 0.62) | 0.88 (0.85 to 0.91) |

NOTE: Pooled estimates and 95% CI across 20 multiply imputed datasets are presented unless otherwise stated. (NOTE: 1 - Sensitivity and specificity for formal prediction based on a single imputation with bootstrap 95% CIs fixed at the observed doctors informal sensitivity/specificity; 2 - No calibration was possible since intercepts were not available; and 3 - 95% ZL CIs are provided for doctors measures of accuracy). ABBREVIATIONS: IQR – interquartile range; ORC – ordinal *c*-index; SSV – six simple variables model
