# Supplementary material for: Comparison of Statistical and Clinical Predictions of Functional Outcome after Ischemic Stroke
Source: PLoS One. 2014 Oct 9;9(10):e110189. doi: 10.1371/journal.pone.0110189 (PMC4192583; doi:10.1371/journal.pone.0110189)
Supplement: Table S3 — Prediction of poor outcome (OHS≥3) following stroke for inpatients (N = 442), outpatients (N = 489) and all patients (N = 931). (DOC) [file pone.0110189.s005.doc]

**Table S3 - Prediction of poor outcome (OHS≥3**) following stroke for inpatients (N=442), outpatients (N=489) and all patients (N=931)

|  |  | **Calibration** | |  | **Discrimination** | |  | **Fixed informal sensitivity/specificity^1^** | |
| --- | --- | --- | --- | --- | --- | --- | --- | --- | --- |
| **Patients** | **Median R^2^ (%, IQR)** | **Intercept** | **Slope** |  | **ORC** | **AUROCC** |  | **Sensitivity** | **Specificity** |
| **Inpatients** |  |  |  |  |  |  |  |  |  |
| Reid | 34.7 (34.4 to 34.9) | 0.84 (0.69 to 0.98) | 0.35 (0.31 to 0.39) |  | 0.71 (0.68 to 0.75) | 0.82 (0.78 to 0.86) |  | 0.51 (0.39 to 0.60) | 0.89 (0.82 to 0.94) |
| Weimar | 36.5 (36.1 to 36.8) | 0.77 (0.65 to 0.88) | 0.79 (0.70 to 0.89) |  | 0.71 (0.68 to 0.75) | 0.82 (0.78 to 0.86) |  | 0.51 (0.42 to 0.61) | 0.89 (0.82 to 0.94) |
| SSV | 37.6 (37.3 to 37.7) | -0.11 (-0.24 to 0.02) | 0.61 (0.55 to 0.68) |  | 0.73 (0.70 to 0.76) | 0.82 (0.78 to 0.86) |  | 0.52 (0.42 to 0.63) | 0.90 (0.84 to 0.95) |
| Appelros^2^ | 37.6 (37.4 to 37.8) | - | - |  | 0.72 (0.68 to 0.75) | 0.81 (0.77 to 0.85) |  | 0.52 (0.41 to 0.61) | 0.89 (0.82 to 0.94) |
| Lee^2^ | 24.6 (24.3 to 24.7) | - | - |  | 0.66 (0.62 to 0.71) | 0.75 (0.70 to 0.79) |  | 0.47 (0.37 to 0.54) | 0.84 (0.76 to 0.91) |
| Doctor^3^ | - | - | - |  | 0.72 (0.69 to 0.77) | - |  | 0.55 (0.49 to 0.62) | 0.91 (0.87 to 0.95) |
| **Outpatients** |  |  |  |  |  |  |  |  |  |
| Reid | 12.0 (11.7 to 12.1) | 1.33 (1.19 to 1.46) | 0.68 (0.58 to 0.79) |  | 0.65 (0.61 to 0.70) | 0.71 (0.65 to 0.78) |  | 0.16 (0.10 to 0.30) | 0.99 (0.97 to 1.00) |
| Weimar | 8.8 (8.6 to 9.2) | 0.36 (0.24 to 0.48) | 1.04 (0.82 to 1.26) |  | 0.62 (0.58 to 0.66) | 0.67 (0.60 to 0.73) |  | 0.10 (0.02 to 0.20) | 0.98 (0.95 to 1.00) |
| SSV | 8.6 (8.6 to 8.6) | -0.48 (-0.61 to -0.35) | 0.54 (0.43 to 0.66) |  | 0.60 (0.55 to 0.65) | 0.64 (0.57 to 0.71) |  | 0.15 (0.05 to 0.27) | 0.98 (0.97 to 1.00) |
| Appelros^2^ | 7.0 (6.8 to 7.3) | - | - |  | 0.61 (0.57 to 0.66) | 0.66 (0.59 to 0.73) |  | 0.09 (0.01 to 0.17) | 0.98 (0.94 to 0.99) |
| Lee^2^ | 0.0 (0.0 to 0.1) | - | - |  | 0.57 (0.51 to 0.62) | 0.57 (0.50 to 0.65) |  | 0.07 (0.01 to 0.15) | 0.97 (0.93 to 0.99) |
| Doctor^3^ | - | - | - |  | 0.65 (0.61 to 0.69) | - |  | 0.09 (0.04 to 0.16) | 0.98 (0.96 to 0.99) |
| **All patients** |  |  |  |  |  |  |  |  |  |
| Reid | 41.8 (41.6 to 42.0) | 1.09 (0.98 to 1.19) | 0.46 (0.43 to 0.50) |  | 0.75 (0.73 to 0.77) | 0.84 (0.81 to 0.87) |  | 0.45 (0.34 to 0.52) | 0.96 (0.93 to 0.98) |
| Weimar | 40.0 (39.9 to 40.2) | 0.57 (0.49 to 0.65) | 0.98 (0.90 to 1.06) |  | 0.73 (0.71 to 0.76) | 0.82 (0.79 to 0.85) |  | 0.43 (0.35 to 0.51) | 0.96 (0.92 to 0.98) |
| SSV | 39.2 (39.0 to 39.5) | -0.30 (-0.39 to -0.21) | 0.71 (0.66 to 0.76) |  | 0.72 (0.70 to 0.74) | 0.81 (0.78 to 0.84) |  | 0.43 (0.36 to 0.51) | 0.95 (0.93 to 0.98) |
| Appelros^2^ | 39.6 (39.5 to 39.8) | - | - |  | 0.73 (0.71 to 0.75) | 0.82 (0.79 to 0.85) |  | 0.42 (0.35 to 0.50) | 0.95 (0.93 to 0.97) |
| Lee^2^ | 27.9 (27.7 to 28.0) | - | - |  | 0.69 (0.66 to 0.71) | 0.76 (0.72 to 0.79) |  | 0.38 (0.32 to 0.45) | 0.94 (0.91 to 0.96) |
| Doctor^3^ | - | - | - |  | 0.74 (0.72 to 0.76) | - |  | 0.44 (0.39 to 0.49) | 0.96 (0.94 to 0.97) |

NOTE: Pooled estimates and 95% CI across 20 multiply imputed datasets are presented unless otherwise stated. (NOTE: 1 - Sensitivity and specificity for formal prediction based on a single imputation with bootstrap 95% CIs fixed at the observed doctors informal sensitivity/specificity; 2 - No calibration was possible since intercepts were not available; and 3 - 95% ZL CIs are provided for doctors measures of accuracy). ABBREVIATIONS: IQR – interquartile range; ORC – ordinal *c*-index; SSV – six simple variables model
