# Supplementary material for: Comparison of Statistical and Clinical Predictions of Functional Outcome after Ischemic Stroke
Source: PLoS One. 2014 Oct 9;9(10):e110189. doi: 10.1371/journal.pone.0110189 (PMC4192583; doi:10.1371/journal.pone.0110189)
Supplement: Table S4 — Prediction of poor outcome (OHS≥3) following stroke for patients assessed within: 2 days (N = 197); 2 to 7 days (N = 234) and over 7 days (N = 500) from stroke onset. (DOC) [file pone.0110189.s006.doc]

**Table S4 - Prediction of poor outcome (OHS≥3**) following stroke for patients assessed within: 2 days (N=197); 2 to 7 days (N=234) and over 7 days (N=500) from stroke onset

|  |  | **Calibration** | |  | **Discrimination** | |  | **Fixed informal sensitivity/specificity^1^** | |
| --- | --- | --- | --- | --- | --- | --- | --- | --- | --- |
| **Delay** | **Median R^2^ (%, IQR)** | **Intercept** | **Slope** |  | **ORC** | **AUROCC** |  | **Sensitivity** | **Specificity** |
| **<2 days** |  |  |  |  |  |  |  |  |  |
| Reid | 43.7 (42.8 to 44.6) | 0.12 (-0.13 to 0.36) | 0.36 (0.30 to 0.41) |  | 0.73 (0.68 to 0.77) | 0.85 (0.80 to 0.91) |  | 0.56 (0.42 to 0.78) | 0.86 (0.79 to 0.94) |
| Weimar | 41.5 (40.8 to 42.3) | 0.48 (0.29 to 0.66) | 0.77 (0.65 to 0.89) |  | 0.72 (0.68 to 0.77) | 0.84 (0.79 to 0.90) |  | 0.60 (0.40 to 0.74) | 0.88 (0.78 to 0.95) |
| SSV | 46.9 (46.2 to 47.0) | -0.39 (-0.60 to -0.18) | 0.71 (0.61 to 0.81) |  | 0.72 (0.68 to 0.76) | 0.86 (0.81 to 0.91) |  | 0.68 (0.42 to 0.82) | 0.91 (0.84 to 0.97) |
| Appelros^2^ | 42.0 (41.6 to 42.5) | - | - |  | 0.72 (0.68 to 0.77) | 0.84 (0.78 to 0.89) |  | 0.61 (0.43 to 0.75) | 0.89 (0.78 to 0.95) |
| Lee^2^ | 32.7 (32.4 to 33.4) | - | - |  | 0.66 (0.61 to 0.71) | 0.80 (0.74 to 0.86) |  | 0.54 (0.34 to 0.67) | 0.81 (0.69 to 0.91) |
| Doctor^3^ | - | - | - |  | 0.74 (0.70 to 0.78) | - |  | 0.63 (0.54 to 0.71) | 0.90 (0.82 to 0.95) |
| **2 to 7 days** |  |  |  |  |  |  |  |  |  |
| Reid | 36.6 (36.2 to 36.8) | 0.80 (0.61 to 0.98) | 0.46 (0.39 to 0.53) |  | 0.77 (0.74 to 0.81) | 0.85 (0.81 to 0.90) |  | 0.48 (0.36 to 0.60) | 0.92 (0.82 to 0.97) |
| Weimar | 44.1 (43.9 to 44.3) | 0.58 (0.43 to 0.73) | 1.19 (1.02 to 1.36) |  | 0.76 (0.72 to 0.79) | 0.86 (0.82 to 0.91) |  | 0.51 (0.36 to 0.63) | 0.93 (0.85 to 0.98) |
| SSV | 38.7 (38.7 to 39.5) | -0.33 (-0.50 to -0.16) | 0.65 (0.56 to 0.73) |  | 0.73 (0.69 to 0.77) | 0.84 (0.79 to 0.88) |  | 0.42 (0.32 to 0.57) | 0.89 (0.83 to 0.95) |
| Appelros^2^ | 47.9 (47.8 to 48.0) | - | - |  | 0.75 (0.72 to 0.79) | 0.86 (0.82 to 0.91) |  | 0.50 (0.37 to 0.68) | 0.92 (0.86 to 0.97) |
| Lee^2^ | 24.4 (24.2 to 24.6) | - | - |  | 0.72 (0.68 to 0.76) | 0.73 (0.66 to 0.79) |  | 0.45 (0.34 to 0.55) | 0.88 (0.78 to 0.97) |
| Doctor^3^ | - | - | - |  | 0.74 (0.71 to 0.78) | - |  | 0.49 (0.39 to 0.58) | 0.92 (0.87 to 0.96) |
| **>7 days** |  |  |  |  |  |  |  |  |  |
| Reid | 19.7 (19.3 to 20.0) | 1.57 (1.44 to 1.70) | 0.75 (0.65 to 0.85) |  | 0.74 (0.70 to 0.77) | 0.74 (0.68 to 0.80) |  | 0.22 (0.11 to 0.37) | 0.99 (0.97 to 1.00) |
| Weimar | 15.6 (15.3 to 15.9) | 0.60 (0.49 to 0.72) | 1.13 (0.94 to 1.31) |  | 0.71 (0.67 to 0.75) | 0.68 (0.62 to 0.74) |  | 0.16 (0.08 to 0.25) | 0.97 (0.94 to 1.00) |
| SSV | 15.3 (15.3 to 15.3) | -0.26 (-0.38 to -0.13) | 0.66 (0.55 to 0.76) |  | 0.70 (0.66 to 0.75) | 0.66 (0.60 to 0.73) |  | 0.22 (0.10 to 0.37) | 0.98 (0.97 to 1.00) |
| Appelros^2^ | 11.0 (10.9 to 11.2) | - | - |  | 0.70 (0.67 to 0.75) | 0.67 (0.60 to 0.73) |  | 0.15 (0.07 to 0.23) | 0.97 (0.92 to 0.99) |
| Lee^2^ | 4.0 (3.8 to 4.0) | - | - |  | 0.66 (0.61 to 0.71) | 0.63 (0.55 to 0.70) |  | 0.18 (0.10 to 0.27) | 0.98 (0.95 to 1.00) |
| Doctor^3^ | - | - | - |  | 0.72 (0.68 to 0.76) | - |  | 0.17 (0.10 to 0.25) | 0.98 (0.96 to 0.99) |

Pooled estimates and 95% CI across 20 multiply imputed datasets are presented unless otherwise stated. (NOTE: 1 - Sensitivity and specificity for formal prediction based on a single imputation with bootstrap 95% CIs fixed at the observed doctors informal sensitivity/specificity; 2 - No calibration was possible since intercepts were not available; and 3 - 95% ZL CIs are provided for doctors measures of accuracy). ABBREVIATIONS: IQR – interquartile range; ORC – ordinal *c*-index; SSV – six simple variables model
